# Supplementary material for: UTX is an escape from X-inactivation tumor-suppressor in B cell lymphoma
Source: Nat Commun. 2018 Jul 13;9:2720. doi: 10.1038/s41467-018-05084-w (PMC6045675; doi:10.1038/s41467-018-05084-w)
Supplement: Supplementary file 1 — Supplementary Information [file 41467_2018_5084_MOESM1_ESM.docx]

**UTX is an escape from X-inactivation tumor-suppressor in B cell lymphoma**

**Li *et al.***

**Supplementary information**

**Supplementary tables**

Supplementary Table 1 List of top 30 enriched pathways of DEGs in Eμ-Myc;UTX KO lymphomas

Supplementary Table 2 Up regulated genes(58) in Eμ-Myc;UTX KO lymphomas

Supplementary Table 3 Down regulated genes(62) in Eμ-Myc;UTX KO lymphomas

Supplementary Table 4 List of qPCR primers used in this study

Supplementary Table 5 List of shRNA sequence used in this study

Supplementary Table 6 List of anti-cancer drugs used in this study

**Supplementary figures**

Supplementary Figure 1 Breeding scheme for generation of Eμ-Myc;UTX KO mice

Supplementary Figure 2 Representative surface marker analysis of each stage of B cell differentiation

Supplementary Figure 3 HE staining images of spleen and nodal lymphoma with each genotype

Supplementary Figure 4 Representative images of lymphoma dissemination and enhanced blood vessel formation in Eμ-Myc;UTX KO mice

Supplementary Figure 5 Kaplan-Meier survival curves risk grouping by expression of UTX in male and female group respectively

Supplementary Figure 6 Original western blot data for anti-UTX.

**Supplementary Table 1 List of top 30 enriched pathways of DEGs in Eμ-Myc;UTX KO lymphomas.**

| # | Pathway | DEGs genes* | Differentially expressed genes |
| --- | --- | --- | --- |
| 1 | Primary immunodeficiency | 12 (11.76%) | H2-Eb1, H2-DMa, H2-Aa, H2-DMb2, Rag1, H2-Ab1, H2-Ob, Igll1, H2-DMb1, Ciita, H2-Oa, H2-Q8 |
| 2 | Hematopoietic cell lineage | 13 (12.75%) | H2-Eb1, H2-DMa, Cd36, H2-Aa, H2-DMb2, H2-Ab1, H2-Ob, Dntt, Cd2, H2-DMb1, H2-Oa, Ms4a1, H2-Q8 |
| 3 | Antigen processing and presentation | 11 (10.78%) | Cd74, H2-Eb1, H2-DMa, H2-Aa, H2-DMb2, H2-Ab1, H2-Ob, H2-DMb1, Ciita, H2-Oa, H2-Q8 |
| 4 | Cell adhesion molecules (CAMs) | 16 (15.69%) | H2-Eb1, H2-DMa, H2-Aa, Tmeff1, H2-DMb2, Reln, H2-Ab1, H2-Ob, Cldn4, Fstl1, Cd2, H2-DMb1, Sdc1, H2-Oa, Thy1, H2-Q8 |
| 5 | T cell receptor signaling pathway | 11 (10.78%) | H2-Eb1, H2-DMa, H2-Aa, H2-DMb2, Rasgrp1, H2-Ab1, H2-Ob, Lat, H2-DMb1, H2-Oa, H2-Q8 |
| 6 | Malaria | 5 (4.9%) | Cd36, Hbb-bs, Hba-a2, Hba-a1, Thy1 |
| 7 | ECM-receptor interaction | 5 (4.9%) | Cd36, Tmeff1, Reln, Fstl1, Sdc1 |
| 8 | African trypanosomiasis | 3 (2.94%) | Hbb-bs, Hba-a2, Hba-a1 |
| 9 | Tuberculosis | 5 (4.9%) | Cd74, Cabp4, Camk2b, Ciita, Thy1 |
| 10 | Amphetamine addiction | 3 (2.94%) | Ddc, Cabp4, Camk2b |
| 11 | Histidine metabolism | 2 (1.96%) | Ddc, Aldh1b1 |
| 12 | Glycosaminoglycan biosynthesis -  heparan sulfate / heparin | 2 (1.96%) | Tsc22d1, Wscd1 |
| 13 | Riboflavin metabolism | 1 (0.98%) | Blvrb |
| 14 | Leukocyte transendothelial migration | 5 (4.9%) | Reln, Cldn4, Sdc1, Actn1, Thy1 |
| 15 | Arrhythmogenic right ventricular cardiomyopathy (ARVC) | 4 (3.92%) | Gja1, Reln, Sdc1, Actn1 |
| 16 | Phagosome | 5 (4.9%) | Cd36, Reln, Tubb3, Sdc1, Thy1 |
| 17 | Oxytocin signaling pathway | 4 (3.92%) | Cabp4, Mef2c, Camk2b, Rgs2 |
| 18 | Tryptophan metabolism | 2 (1.96%) | Ddc, Aldh1b1 |
| 19 | Phototransduction - fly | 2 (1.96%) | Cabp4, Camk2b |
| 20 | cGMP-PKG signaling pathway | 4 (3.92%) | Cabp4, Mef2c, Gtf2ird1, Rgs2 |
| 21 | Axon guidance | 5 (4.9%) | Efnb1, Reln, Bcar3, Camk2b, Sdc1 |
| 22 | Pertussis | 4 (3.92%) | Cabp4, Reln, Sdc1, Thy1 |
| 23 | Tight junction | 4 (3.92%) | Prkch, Uaca, Cldn4, Actn1 |
| 24 | FoxO signaling pathway | 3 (2.94%) | S1pr1, Rag1, Plk2 |
| 25 | Pathogenic Escherichia coli infection | 4 (3.92%) | Reln, Tubb3, Cldn4, Sdc1 |
| 26 | Prion diseases | 2 (1.96%) | Tmeff1, Fstl1 |
| 27 | Inflammatory mediator regulation of TRP channels | 3 (2.94%) | Cabp4, Camk2b, Prkch |
| 28 | Dopaminergic synapse | 3 (2.94%) | Ddc, Cabp4, Camk2b |
| 29 | Lysine degradation | 2 (1.96%) | Zcwpw1, Aldh1b1 |
| 30 | Non-homologous end-joining | 1 (0.98%) | Dntt |

*** DEGs genes with pathway annotation (102)Supplementary Table 2 Up regulated genes in Eμ-Myc;UTX KO lymphomas.**

| GeneID | log2Ratio (KO/WT) | Symbol | Description |
| --- | --- | --- | --- |
| 102465598 | 9.24 | Mir6989 | microRNA 6989 |
| 18546 | 8.38 | Pcp4 | Purkinje cell protein 4 |
| 230157 | 7.59 | Tmeff1 | transmembrane protein with EGF-like and two follistatin-like domains 1 |
| 13641 | 6.99 | Efnb1 | ephrin B1 |
| 14609 | 6.20 | Gja1 | gap junction protein, alpha 1 |
| 14314 | 6.19 | Fstl1 | follistatin-like 1 |
| 29815 | 6.07 | Bcar3 | breast cancer anti-estrogen resistance 3 |
| 69169 | 6.05 | Fcmr | Fc fragment of IgM receptor |
| 19373 | 6.04 | Rag1 | recombination activating gene 1 |
| 66972 | 5.88 | Slc25a23 | solute carrier family 25 (mitochondrial carrier; phosphate carrier), member 23 |
| 12143 | 5.72 | Blk | B lymphoid kinase |
| 19699 | 5.55 | Reln | reelin |
| 70427 | 5.50 | Mier2 | MIER family member 2 |
| 93721 | 5.48 | Cpn1 | carboxypeptidase N, polypeptide 1 |
| 15000 | 5.42 | H2-DMb2 | histocompatibility 2, class II, locus Mb2 |
| 381678 | 5.36 | Zcwpw1 | zinc finger, CW type with PWWP domain 1 |
| 100628611 | 5.32 | Mir5107 | microRNA 5107 |
| 16149 | 4.98 | Cd74 | CD74 antigen (invariant polypeptide of major histocompatibility complex, class II antigen-associated) |
| 14969 | 4.91 | H2-Eb1 | histocompatibility 2, class II antigen E beta |
| 20568 | 4.91 | Slpi | secretory leukocyte peptidase inhibitor |
| 242248 | 4.84 | Bank1 | B cell scaffold protein with ankyrin repeats 1 |
| 100039257 | 4.73 | Tmem254b | transmembrane protein 254b |
| 14960 | 4.65 | H2-Aa | histocompatibility 2, class II antigen A, alpha |
| 12491 | 4.54 | Cd36 | CD36 antigen |
| 14998 | 4.52 | H2-DMa | histocompatibility 2, class II, locus DMa |
| 12481 | 4.44 | Cd2 | CD2 antigen |
| 15019 | 4.29 | H2-Q8 | histocompatibility 2, Q region locus 8 |
| 107771 | 4.25 | Bmyc | brain expressed myelocytomatosis oncogene |
| 19735 | 4.09 | Rgs2 | regulator of G-protein signaling 2 |
| 14999 | 4.05 | H2-DMb1 | histocompatibility 2, class II, locus Mb1 |
| 67155 | 3.77 | Smarca2 | SWI/SNF related, matrix associated, actin dependent regulator of chromatin, subfamily a, member 2 |
| 12265 | 3.63 | Ciita | class II transactivator |
| 21807 | 3.62 | Tsc22d1 | TSC22 domain family, member 1 |
| 140488 | 3.61 | Igf2bp3 | insulin-like growth factor 2 mRNA binding protein 3 |
| 15001 | 3.27 | H2-Oa | histocompatibility 2, O region alpha locus |
| 15122 | 3.15 | Hba-a1 | hemoglobin alpha, adult chain 1 |
| 69202 | 3.03 | Ptms | parathymosin |
| 20620 | 2.96 | Plk2 | polo-like kinase 2 |
| 209086 | 2.95 | Samd9l | sterile alpha motif domain containing 9-like |
| 14961 | 2.90 | H2-Ab1 | histocompatibility 2, class II antigen A, beta 1 |
| 233016 | 2.86 | Blvrb | biliverdin reductase B (flavin reductase (NADPH)) |
| 14238 | 2.77 | Foxf2 | forkhead box F2 |
| 106389 | 2.76 | Eaf2 | ELL associated factor 2 |
| 100503605 | 2.67 | Hbb-bs | hemoglobin, beta adult s chain |
| 13609 | 2.66 | S1pr1 | sphingosine-1-phosphate receptor 1 |
| 12482 | 2.58 | Ms4a1 | membrane-spanning 4-domains, subfamily A, member 1 |
| 76933 | 2.53 | Ifi27l2a | interferon, alpha-inducible protein 27 like 2A |
| 20195 | 2.50 | S100a11 | S100 calcium binding protein A11 |
| 19419 | 2.46 | Rasgrp1 | RAS guanyl releasing protein 1 |
| 12798 | 2.44 | Cnn2 | calponin 2 |
| 208154 | 2.40 | Btla | B and T lymphocyte associated |
| 23833 | 2.40 | Cd52 | CD52 antigen |
| 98752 | 2.39 | Fcrla | Fc receptor-like A |
| 15002 | 2.36 | H2-Ob | histocompatibility 2, O region beta locus |
| 110257 | 2.35 | Hba-a2 | hemoglobin alpha, adult chain 2 |
| 17260 | 2.29 | Mef2c | myocyte enhancer factor 2C |
| 13036 | 2.14 | Ctsh | cathepsin H |
| 17068 | 2.02 | Ly6d | lymphocyte antigen 6 complex, locus D |

**Supplementary Table 3 Down regulated genes in Eμ-Myc;UTX KO lymphomas.**

| GeneID | log2Ratio (KO/WT) | Symbol | Description |
| --- | --- | --- | --- |
| 21673 | -11.81 | Dntt | deoxynucleotidyltransferase, terminal |
| 22363 | -10.51 | Vpreb2 | pre-B lymphocyte gene 2 |
| 382077 | -9.79 | Ccdc33 | coiled-coil domain containing 33 |
| 100316682 | -9.35 | Mir1892 | microRNA 1892 |
| 216881 | -8.76 | Wscd1 | WSC domain containing 1 |
| 13195 | -8.21 | Ddc | dopa decarboxylase |
| 12705 | -7.57 | Cited1 | Cbp/p300-interacting transactivator with Glu/Asp-rich carboxy-terminal domain 1 |
| 231633 | -7.51 | Tmem119 | transmembrane protein 119 |
| 268515 | -6.57 | Bahcc1 | BAH domain and coiled-coil containing 1 |
| 75141 | -6.42 | Rasd2 | RASD family, member 2 |
| 16136 | -6.11 | Igll1 | immunoglobulin lambda-like polypeptide 1 |
| 114128 | -6.01 | Laptm4b | lysosomal-associated protein transmembrane 4B |
| 18755 | -5.90 | Prkch | protein kinase C, eta |
| 791347 | -5.38 | Gm10007 | predicted gene 10007 |
| 22152 | -5.26 | Tubb3 | tubulin, beta 3 class III |
| 12740 | -5.24 | Cldn4 | claudin 4 |
| 14585 | -5.20 | Gfra1 | glial cell line derived neurotrophic factor family receptor alpha 1 |
| 22362 | -5.19 | Vpreb1 | pre-B lymphocyte gene 1 |
| 269295 | -5.13 | Rtn4rl2 | reticulon 4 receptor-like 2 |
| 73660 | -4.90 | Cabp4 | calcium binding protein 4 |
| 74410 | -4.88 | Ttll11 | tubulin tyrosine ligase-like family, member 11 |
| 57080 | -4.86 | Gtf2ird1 | general transcription factor II I repeat domain-containing 1 |
| 72709 | -4.81 | C1qtnf6 | C1q and tumor necrosis factor related protein 6 |
| 16797 | -4.39 | Lat | linker for activation of T cells |
| 51800 | -4.27 | Bok | BCL2-related ovarian killer |
| 74100 | -4.12 | Arpp21 | cyclic AMP-regulated phosphoprotein, 21 |
| 72565 | -4.11 | Uaca | uveal autoantigen with coiled-coil domains and ankyrin repeats |
| 21838 | -4.08 | Thy1 | thymus cell antigen 1, theta |
| 319710 | -4.06 | Frmd6 | FERM domain containing 6 |
| 109711 | -4.03 | Actn1 | actinin, alpha 1 |
| 68713 | -3.95 | Ifitm1 | interferon induced transmembrane protein 1 |
| 12323 | -3.76 | Camk2b | calcium/calmodulin-dependent protein kinase II, beta |
| 80876 | -3.69 | Ifitm2 | interferon induced transmembrane protein 2 |
| 72685 | -3.67 | Dnajc6 | DnaJ heat shock protein family (Hsp40) member C6 |
| 17750 | -3.66 | Mt2 | metallothionein 2 |
| 110279 | -3.62 | Bcr | breakpoint cluster region |
| 56473 | -3.58 | Fads2 | fatty acid desaturase 2 |
| 381148 | -3.41 | Prob1 | proline rich basic protein 1 |
| 83554 | -3.29 | Fstl3 | follistatin-like 3 |
| 13106 | -3.28 | Cyp2e1 | cytochrome P450, family 2, subfamily e, polypeptide 1 |
| 77717 | -3.27 | 6030408B16Rik | RIKEN cDNA 6030408B16 gene |
| 29856 | -3.26 | Smtn | smoothelin |
| 20969 | -3.21 | Sdc1 | syndecan 1 |
| 21390 | -3.21 | Tbxa2r | thromboxane A2 receptor |
| 72535 | -3.13 | Aldh1b1 | aldehyde dehydrogenase 1 family, member B1 |
| 85029 | -3.08 | Rpph1 | ribonuclease P RNA component H1 |
| 17341 | -3.08 | Bhlha15 | basic helix-loop-helix family, member a15 |
| 13809 | -2.85 | Enpep | glutamyl aminopeptidase |
| 17748 | -2.81 | Mt1 | metallothionein 1 |
| 328795 | -2.79 | Ubash3a | ubiquitin associated and SH3 domain containing, A |
| 217071 | -2.72 | Gm525 | predicted gene 525 |
| 13876 | -2.72 | Erg | avian erythroblastosis virus E-26 (v-ets) oncogene related |
| 235431 | -2.70 | Coro2b | coronin, actin binding protein, 2B |
| 50873 | -2.64 | Park2 | Parkinson disease (autosomal recessive, juvenile) 2, parkin |
| 545260 | -2.58 | Arsi | arylsulfatase i |
| 14201 | -2.43 | Fhl3 | four and a half LIM domains 3 |
| 67784 | -2.42 | Plxnd1 | plexin D1 |
| 217430 | -2.36 | Pqlc3 | PQ loop repeat containing |
| 66141 | -2.34 | Ifitm3 | interferon induced transmembrane protein 3 |
| 66214 | -2.34 | Rgcc | regulator of cell cycle |
| 53626 | -2.25 | Insm1 | insulinoma-associated 1 |
| 69816 | -1.86 | Mzb1 | marginal zone B and B1 cell-specific protein 1 |

**Supplementary Table 4 List of qPCR primers used in this study.**

| Genes | Sequence |
| --- | --- |
| Vpreb1 mmu Fwd | 5‘-GCTGCTGGCCTATCTCACAG-3’ |
| Vpreb1 mmu Rev | 5‘-CCAATGTTATGGTCGTTGCTCA-3’ |
| Igll1 mmu Fwd | 5‘-AGCTCAGCAGAAAGGAGCAG-3’ |
| Igll1 mmu Rev | 5‘-GTGGGATGATCTGGAACAGG-3’ |
| Fcmr mmu Fwd | 5‘-CTTCATGAGCAAAGGACACG-3’ |
| Fcmr mmu Rev | 5‘-AAGGTCGGAATCAGGATGTG-3’ |
| Dntt mmu Fwd | 5‘-ACAACTCGAAGAGCCTTCCTC-3’ |
| Dntt mmu Rev | 5‘-GGGTGACCGAATCACTGAGC-3’ |
| Rag1 mmu Fwd | 5‘-AGAGGGGAAACCTTACCTAGAAC-3’ |
| Rag1 mmu Rev | 5‘-TGCTCGTTGAGTCAGAATTGAG-3’ |
| Blk mmu Fwd | 5‘-GAGGCAGGTCAGTGAGAAGG-3’ |
| Blk mmu Rev | 5‘-GTCCTGGTTAGGAGATGGTGG-3’ |
| Bok mmu Fwd | 5‘-AGGTAGTGTCCCTGTATTCCG-3’ |
| Bok mmu Rev | 5‘-AAGGTCTTGCGTACAAACTCC-3’ |
| Efnb1 mmu Fwd | 5‘-TGTGGCTATGGTCGTGCTG-3’ |
| Efnb1 mmu Rev | 5‘-TCTTCGGGTAGATCACCAAGC-3’ |
| Gapdh mmu Fwd | 5‘-AGGTCGGTGTGAACGGATTTG-3’ |
| Gapdh mmu Rev | 5‘-GGGGTCGTTGATGGCAACA-3’ |
| Kdm6a mmu Fwd | 5‘-CCTCATAACCGCACAAACCT-3’ |
| Kdm6a mmu Rev | 5‘-GGACCTGCCAAATGTGAACT-3’ |

**Supplementary Table 5 list of shRNA sequence used in this study.**

| Gene | Species | shRNA | Guide | mRNA target site |
| --- | --- | --- | --- | --- |
| Utx | MOUSE | shUtx-1-1395 | TCTACAGGCAATCTATTGATAA | ATTTATCAATAGATTGCCT |
| Utx | MOUSE | shUtx-2-3921 | TAAACCTTGAAGATCTATATGA | ATTCATATAGATCTTCAAG |

**Supplementary Table 6 List of anti-cancer drugs used in this study.**

| Drug Name | Abbreviation | Mechanism of Action | Drug Category | Concentration Used* |
| --- | --- | --- | --- | --- |
| Gemcitabine | GEM | nucleic acid syn.inhibition | Antimetabolite | 100 nM |
| Cytarabine | Ara-C | nucleic acid syn.inhibition | Antimetabolite | 200 nM |
| 5-Fluorouracil | 5-FU | nucleic acid syn.inhibition | Antimetabolite | 30.5 nM |
| Methotrexate | MTX | nucleic acid syn.inhibition | Antimetabolite | 33 nM |
| Actinomycin D | ActD | RNA syn. Inhibition | Anti-tumor antibiotic | 40 nM |
| 6-Thioguanine | 6-TG | DNA methylation memetic | Antimetabolite | 54 nM |
| Cisplatin | CDDP | DNA crosslinking | Platinum | 3.3 uM |
| Camptothecin | CPT | Induction of SSBs | Topo I poison | 0.4 nM |
| Doxorubicin | DOX | Induction of DSBs | Topo II poison | 16.2 nM |
| Vincristine | VCR | Disruption of mitosis | Antimicrotuble agent | 1.5 nM |
| Pacitaxel | Taxol | Disruption of mitosis | Antimicrotuble agent | 8 nM |
| Decitabine | DAC | DNMT inhibition | Antimetabolite | 0.6 uM |
| Vorinostat | SAHA | HDAC inhibition | HDAC inhibitor | 0.2 uM |
| Vismodegib | GDC-0449 | Hedgehog inhibition | Hedgehog inhibitor | 16 uM |
| Erlotinib | OSI-744 | EGFR inhibition | EGFR inhibitor | 6.5 uM |
| Palbociclib | PD-0332991 | CDK inhibition | Kinase inhibitor | 15 uM |
| Torkinib | PP242 | mTOR inhibition | mTOR inhibitor | 24 uM |
| MK-2206 | MK-2206 | AKT inhibition | AKT inhibitor | 25 uM |
| Olaparib | AZD2281 | Parp1 inhibition | PARP inhibitor | 0.5 uM |
| KU-55933 | KU-55933 | ATM inhibition | ATM inhibitor | 5 uM |
| Bortezomib | PS341 | Proteasome inhibition | Proteosome inhibitor | 13 nM |
| All-trans retinoic acid | ATRA | Cell differentiation | Cell differentiation agent | 25 uM |
| 3-methyladenine | 3-MA-34 | Autophagy inhibitor | Autophagy inhibitor | 2 uM |
| Lovastatin | LOVA | Cholesterol lowering | HMG-CoA Reductase inhibitor | 8.3 uM |

* Listed are concentrations of drugs that cause between 80 and 90% of maximal killing (lethal dose 80-90). For every set of experiments, cell death was monitored using uninfected cells to ensure proper LD80-90s.**
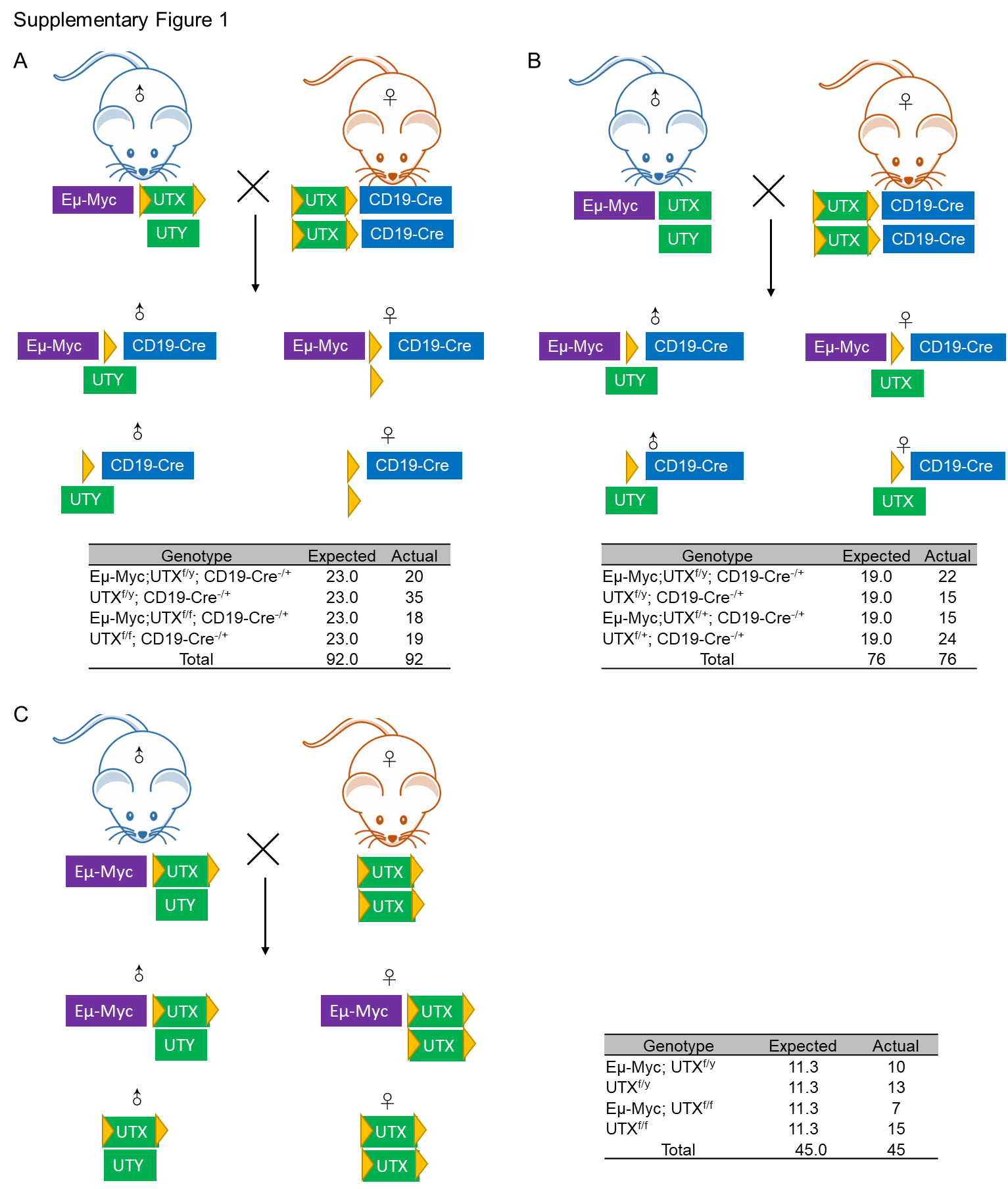
**

**Supplementary Figure 1 Breeding scheme for generation of Eμ-Myc;UTX KO mice**

(A) Breeding scheme for generation of the Eμ-Myc;UTX^f/y^;CD19-Cre^+/-^ and Eμ-Myc;UTX^f/f^;CD19-Cre^+/-^ mice. (B) Breeding scheme for generation of the Eμ-Myc;UTX^f/wt^;CD19-Cre^+/-^ mice. (C) Breeding scheme for generation of the Eμ-Myc;UTX^f/y^ and Eμ-Myc;UTX^f/f^ mice.

**
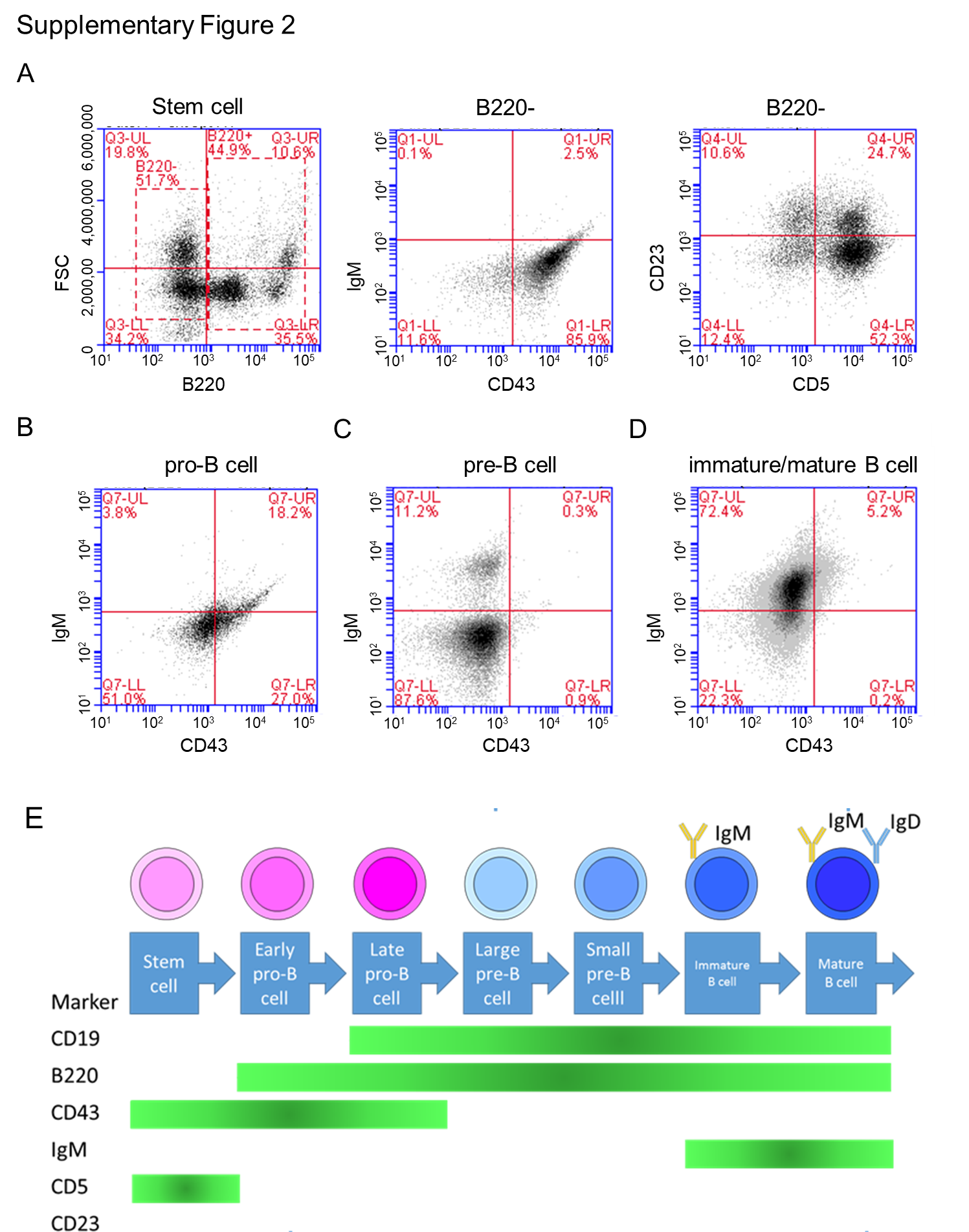
**

**Supplementary Figure 2 Representative surface marker analysis of each stage of B cell differentiation**

(A-D) Representative surface marker analysis indicating stem cell (A), pro-B cell (B), pre-B cell (C) and immature/mature B cell (D). (E) Surface markers during B cell differentiation.


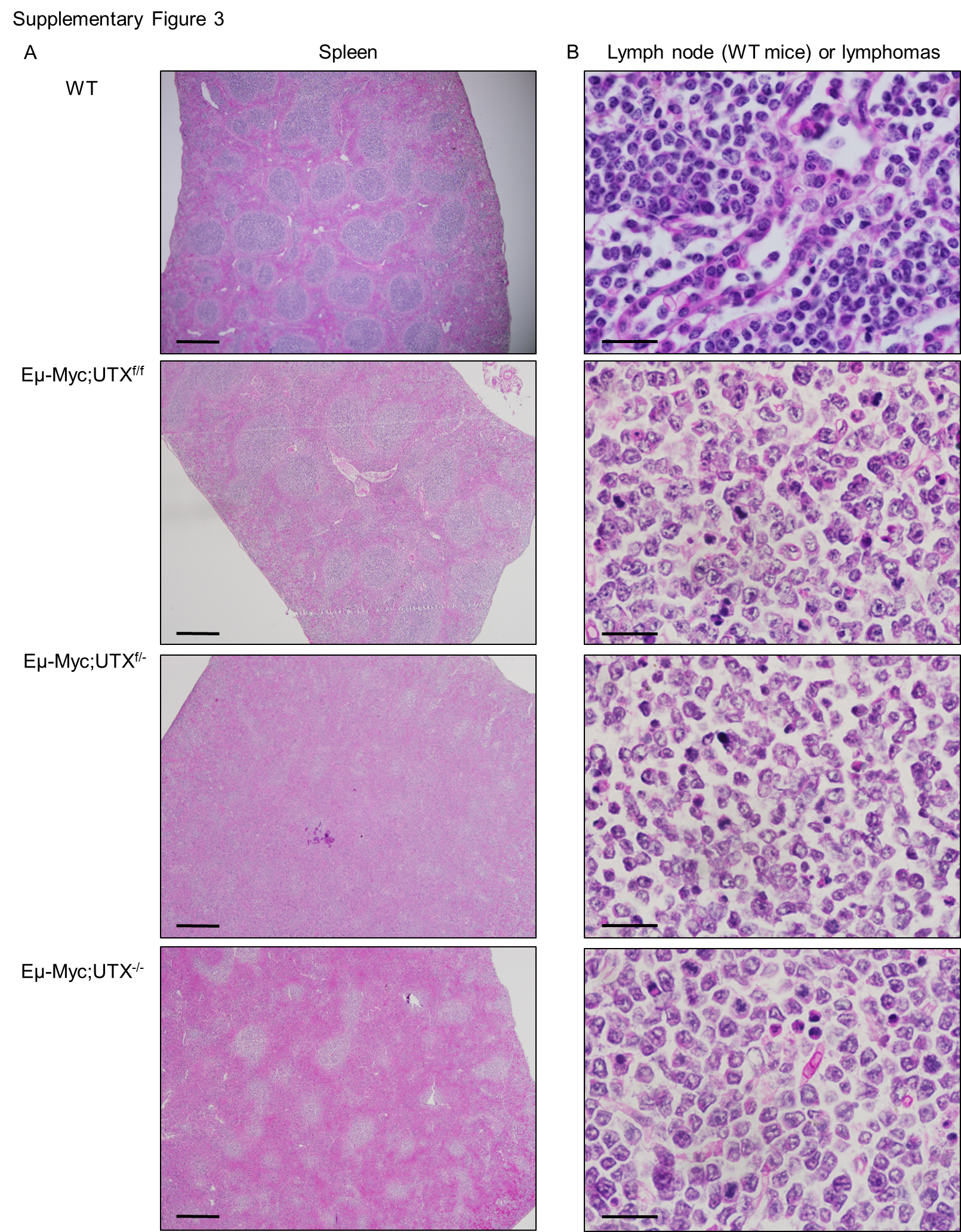


**Supplementary Figure 3 HE staining images of spleen and nodal lymphoma with each genotype**

(A-B) Representative HE staining images of spleen (A) and nodal lymphoma (B) with indicated genotypes. *Bar* in A, 500μm, *Bar* in B, 50μm.


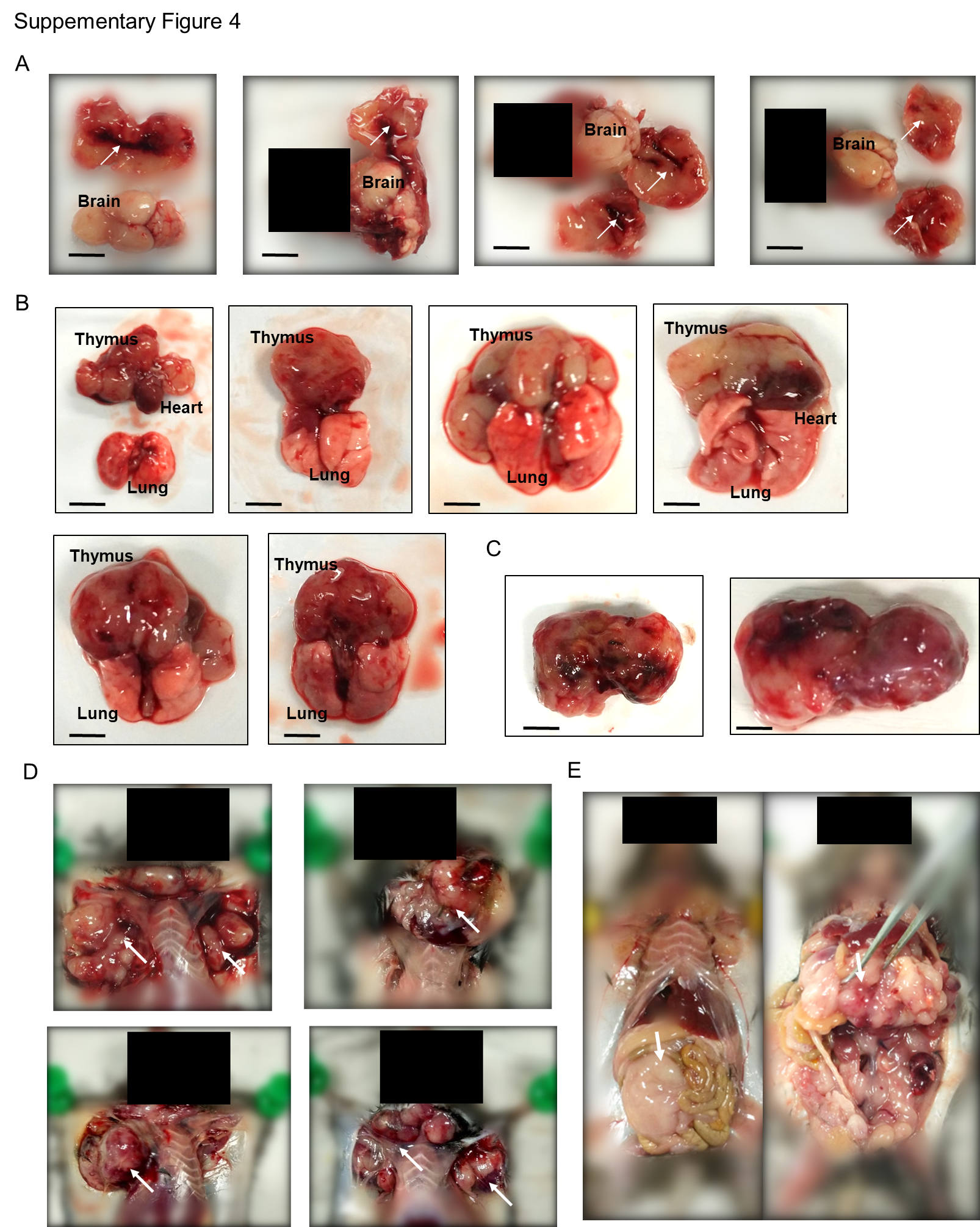


**Supplementary Figure 4 Representative images of lymphoma dissemination and enhanced blood vessel formation in Eμ-Myc;UTX KO mice**

(A) Representative images of lymphoma brain metastasis in UTX KO mice. Arrows indicate lymphomas under skull, which were characterized as B lymphomas by surface marker analysis. (B) Representative images of enlarged thymus in UTX KO mice, and cells from the thymus were characterized as B lymphomas by surface marker analysis. (C,D) Representative images of lymphomas with blood vessel formation in UTX KO mice. (E) Extranodal lymphomas in the gastrointestinal (GI) tract in UTX KO mice. *Bar* in A-C, 5mm.
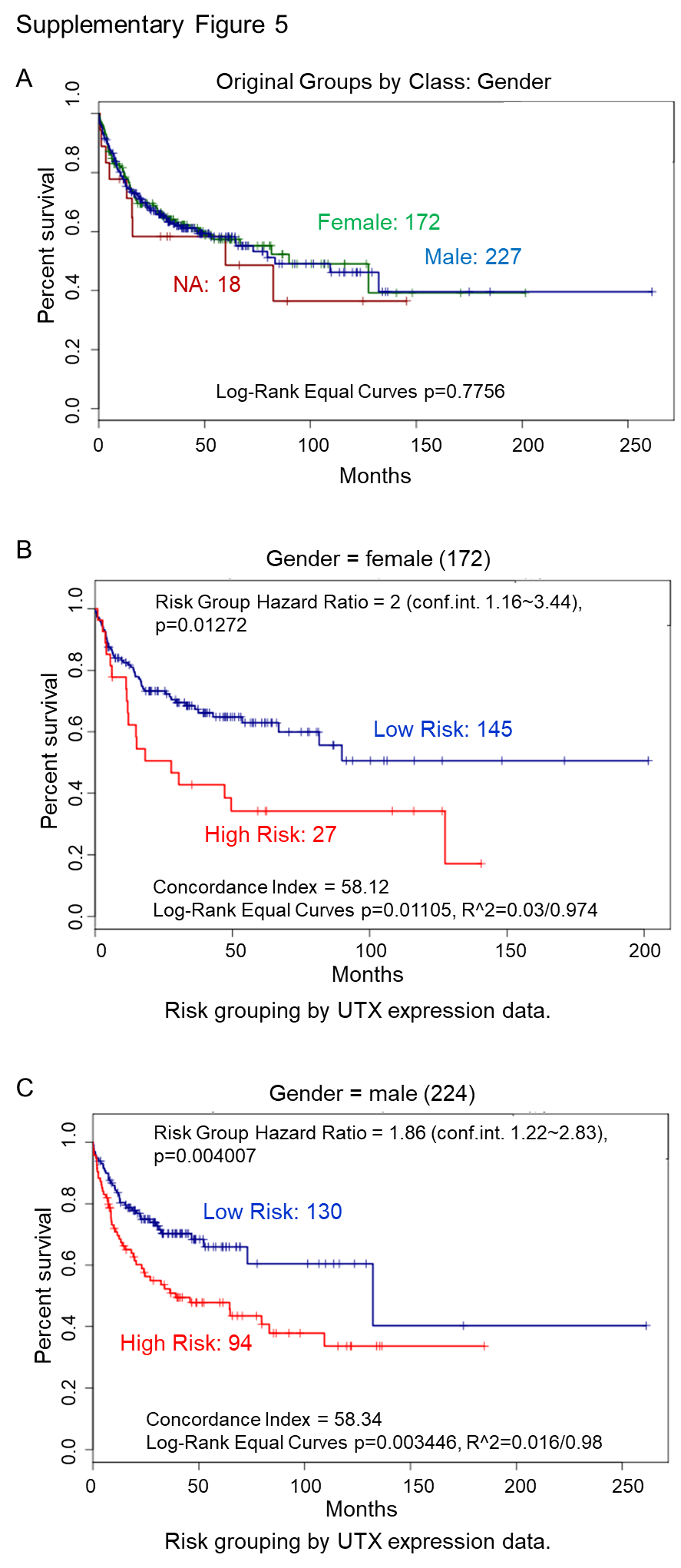


**Supplementary Figure 5 Kaplan-Meier survival curves risk grouping by expression of UTX in male and female group respectively**

(A) Kaplan-Meier survival curves risk grouping by gender. Male, n = 227, Female, n = 172, NA, n = 18. (B-C) Kaplan-Meier survival curves risk grouping by expression of UTX in female (B) and male (C) group respectively. Blue and red lines indicated low-risk and high-risk groups, respectively. Risk grouping was conducted through an optimization algorithm. Analysis was performed by SurvExpress.


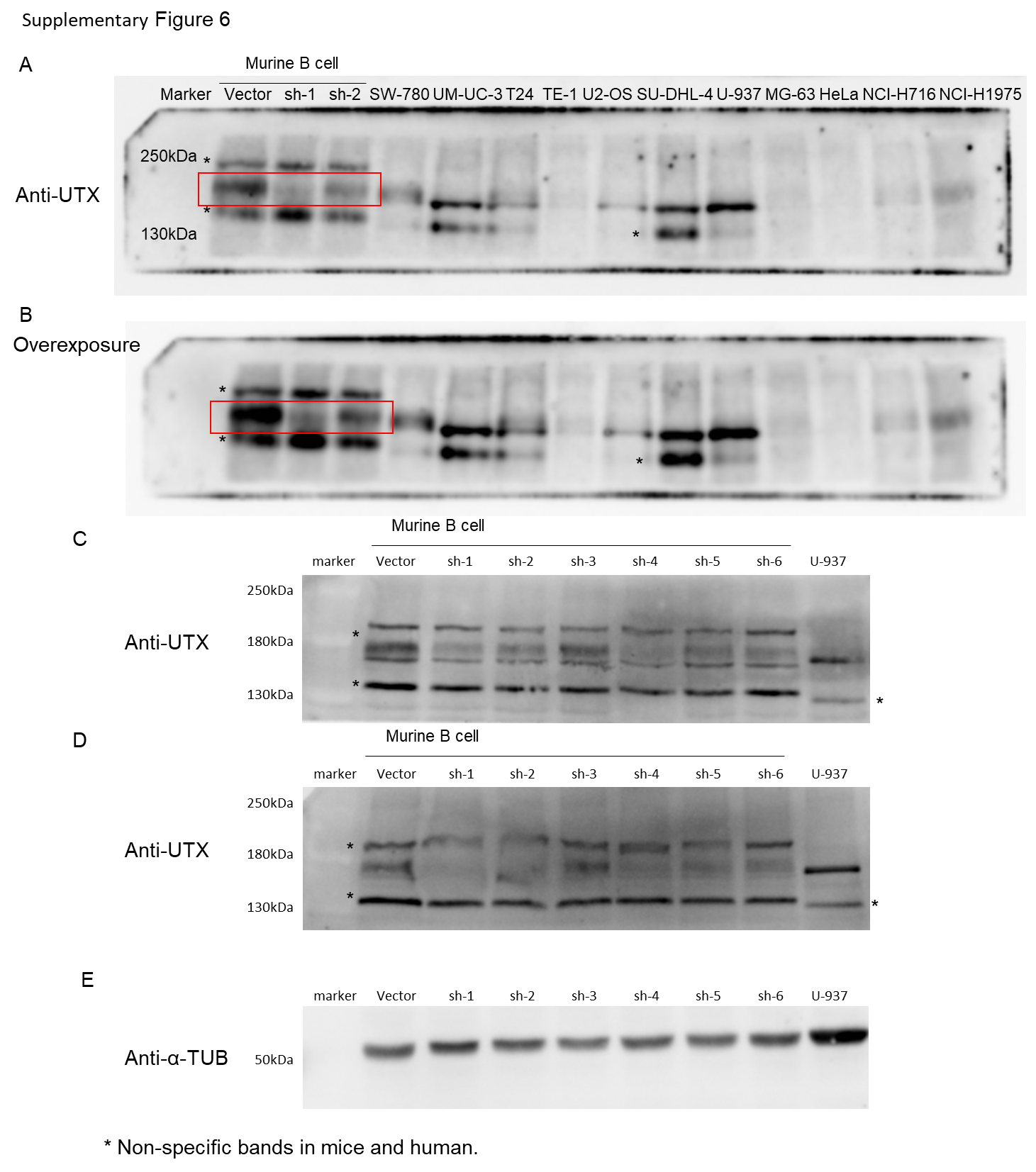


**Supplementary Figure 6 Original western blot data for anti-UTX.**

(A-B) show the uncropped bolts for anti-UTX, * show the non-specific bands, the small band appearing in human cell lysates was reported in many studies using same antibody, the large band appearing in murine B cell lysates was first reported in this study. (C-D) show another two independent repeat experiments to confirm the UTX band in murine B cell, sh-1-6 are six independent shRNA targeting murine Utx, the human cell line U-937 are using as reference, * show the non-specific bands. (E) show internal control to (C-D) using anti-α-TUB.
